# The nitrergic mechanism of geraniol in PTZ-induced seizures

**DOI:** 10.1016/j.ibneur.2025.07.004

**Published:** 2025-07-12

**Authors:** Babak Shahhosseini, Hossein Tahmasebi Dehkordi, Hossein Amini-Khoei, Antoni Sureda, Mehrdad Shahrani, Zahra Lorigooini

**Affiliations:** a Student Research Committee, Shahrekord University of Medical Sciences, Shahrekord, Iran; b Medical Plants Research Center, Basic Health Sciences Institute, Shahrekord University of Medical Sciences, Shahrekord, Iran; c Research Group on Community Nutrition & Oxidative Stress, University of the Balearic Islands-IUNICS, Palma de Mallorca 07122, Spain; d CIBEROBN (Physiopathology of Obesity and Nutrition), Instituto de Salud Carlos III, Madrid E-28029, Spain

**Keywords:** Epilepsy, Seizure, Geraniol, Nitric oxide, Pentylenetetrazole, Nitric oxide synthase

## Abstract

**Background:**

This investigation aims to elucidate the role of NO in the anticonvulsant effects of Geraniol (GER) using a mouse model of pentylenetetrazole (PTZ)-induced seizures.

**Methods:**

Mice were allocated into ten groups, including a control group receiving normal saline. The treatment groups received GER (10, 20, 30, and 40 mg/kg), L-NAME (10 mg/kg), L-arginine (L-arg) at 150 mg/kg, a sub-effective dose of GER (10 mg/kg) combined with L-NAME, and an effective dose of GER (40 mg/kg) plus L-arg, respectively. All drugs were administered *intraperitoneally* 30 min before seizure induction by *intravenous* infusion of PTZ. The last group served as the control for biochemical and molecular tests (no seizure induction). Subsequently, the seizure threshold was recorded. Nitrite levels in serum and the prefrontal cortex (PFC), as well as the gene expression of nNOS and iNOS in the PFC, were assessed.

**Results:**

GER prolonged the seizure threshold and reduced serum and PFC nitrite levels. Also, it downregulated the gene expression of *nNOS* and *iNOS*. Simultaneous administration of L-arg with the effective GER dose (40 mg/kg) notably reversed the beneficial effects of GER. Conversely, when administered with a sub-effective dose of GER (10 mg/kg), L-NAME potentiated the effects of this dose of GER. The expression of the *nNOS* gene in the PFC significantly increased following the administration of 20 mg/kg GER and L-arg plus 40 mg/kg GER. Conversely, 40 mg/kg GER alone reduced *nNOS* gene expression in the PFC.

**Conclusion:**

GER exhibits anticonvulsant properties by modulating the nitrergic system, increasing seizure latency, and reducing NO production. This suggests its potential as a therapeutic candidate for seizure management.

## Graphical Abstract

**Figure:**
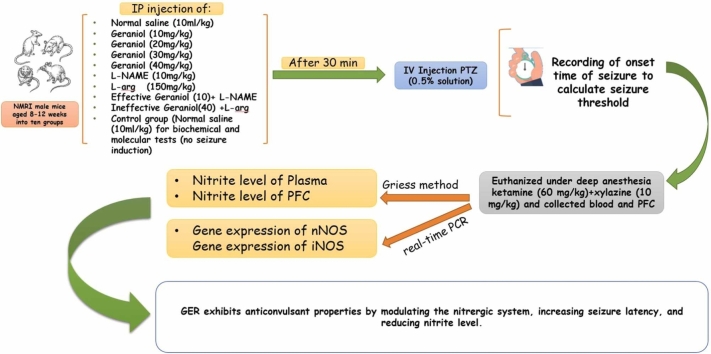

## 1 Introduction

Epileptic seizures, arising from unusual and uncontrolled neuron discharges, involve a spectrum of neurological disorders with diverse causes and manifestations (Fisher et al., 2014). These seizures, whether triggered by factors like electrolyte imbalances, toxins, or injuries or unprovoked, pose a significant global health burden. The incidence rate of epilepsy is estimated at around 50–60 per 100,000 person-years, and up to 8 % of people experience at least one seizure in their lifetime (Asadi-Pooya, 2023, Zhibin Chen et al., 2023). Despite the prevalence of epilepsy, its management remains challenging, with approximately 33 % of patients exhibiting inadequate responses to conventional antiepileptic drugs (AEDs), leading to compromised quality of life (Wahab, 2010).

Recognition of the limitations of existing treatments has led researchers to focus more on the need for new or complementary therapeutic approaches. Hence, this study explores the potential therapeutic role of geraniol (GER) in pentylenetetrazole (PTZ)-induced seizures in mice. GER, a natural monoterpene found in aromatic plants (mainly in citronella rose and palmarosa oils), has gathered attention for its diverse pharmacological effects, including anti-inflammatory, antioxidant, and neuroprotective properties (Ibrahim et al., 2015, Lei et al., 2019, Mączka et al., 2020, Pavan et al., 2018). Markedly, GER inhibits enzymes involved in inflammation, such as cyclooxygenase and nitric oxide synthase (NOS), highlighting its potential in modulating the nitrergic system (Medicherla et al., 2015).

The nitric oxide (NO) pathway plays a key role in seizure pathophysiology. NO, produced by various NOS isoforms (neuronal, endothelial, and inducible), regulates cerebral blood flow, neuronal transmission, memory, and neuroendocrine function (Giorgi et al., 2008). However, increased NO production is observed in seizures, inflammatory disorders, and neurological disabilities (Kovács et al., 2009). Understanding the intricate relationship between NO and seizures, as well as GER's potential, is vital for unraveling GER's anticonvulsant mechanisms (Calabrese et al., 2007, Szabó, 1996).

Indeed, PTZ-induced changes in neuronal nitric oxide synthase (nNOS, also known as Nos1) and inducible nitric oxide synthase (iNOS, also known as Nos2) gene expression highlight the complex role of the nitrergic system in seizures (Kovács et al., 2009). Therefore, this study aimed to investigate the effect of co-administration of L-arginine (L-arg) as a NO precursor and L-N(G)-Nitro arginine methyl ester (L-NAME) as a NOS inhibitor with GER. Furthermore, by analyzing how GER affects the expression of neuronal nitric oxide synthase (*nNOS*), inducible nitric oxide synthase (*iNOS*), and NO levels during PTZ-induced clonic seizures, the study also aimed to fill the gap regarding the potential mechanisms underlying the anticonvulsant properties of GER and pave the way for therapeutic advances.

## 2 Materials and methods

### 2.1 Animals

The experimental procedures involving Naval Medical Research Institute (NMRI) mice were conducted under the guidelines established by Shahrekord University of Medical Sciences. The method is followed by the National Institutes of Health Guide for the Care and Use of Laboratory Animals (8th edition, National Academies Press). Every effort was made to reduce the number of animals used while enhancing their welfare. All protocols were approved by the Ethics Committee of Shahrekord University of Medical Sciences (Reference: IR.SKUMS.REC.1397.332). Moreover, the study was designed, conducted, and reported following the ARRIVE (Animal Research: Reporting of In Vivo Experiments) guidelines to ensure transparency, reproducibility, and adherence to ethical standards.

All animals were maintained under standard laboratory conditions, including a temperature of 22 ± 2°C, a relative humidity of 50 ± 10 %, and a 12:12-h light/dark cycle. The mice were housed in polycarbonate cages on standard bedding material, with no more than four mice per cage to reduce stress and aggression. Mice had free access to standard pellet feed and tap water.

### 2.2 Study design

**Fig. 1:**
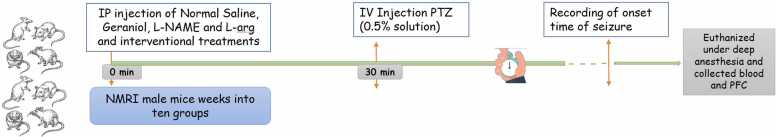
Schematic representation of the experimental protocol.

Male NMRI mice were divided into ten groups: The first group received normal saline (10 ml/kg), while the second to fifth groups received GER (Aldrich-163333) at doses of 10, 20, 30, and 40 mg/kg. The sixth group received L-arg (Sigma-Aldrich-A8094), a NO precursor, at 150 mg/kg, and the seventh group received L-NAME (Sigma-Aldrich-N5751), a NOS inhibitor, at 10 mg/kg. The eighth group received a sub-effective dose of GER (10 mg/kg) plus L-NAME, and the ninth group received an effective dose of GER (40 mg/kg) plus L-arg. The tenth group served as the control for biochemical and molecular tests (no seizure induction). Fig. 1 illustrates a schematic representation of the experimental protocol. GER was dissolved in normal saline with 0.1 % dimethyl sulfoxide (DMSO); all control groups received saline with 0.1 % DMSO. L-NAME and L-arg were administered in normal saline. Doses of GER (10, 20, 30, and 40 mg/kg), L-NAME (10 mg/kg), and L-arg (150 mg/kg) were selected based on previous studies demonstrating their anticonvulsant and neuroprotective properties in rodent models and a pilot study (Amini-Khoei et al., 2018, Hacke et al., 2021, Hosseini et al., 2020). Since this study was designed as an interventional pharmacological investigation, effective and sub-effective doses of GER were selected based on our pilot study; these doses were then optimized to observe both behavioral and biochemical effects. Specifically, 10 mg/kg was considered a sub-effective dose due to the lack of significant change in seizure threshold. In contrast, 40 mg/kg was chosen as the effective dose as it significantly increased the seizure threshold. Based on these results, this research was planned as an interventional study with NO mediators.

All drugs were injected *intraperitoneally* (*ip*) 30 min before the induction of seizures. The total sample size for each group was calculated to be 6–8 for seizure threshold assessment and 3–4 for biochemical and molecular experiments. Subsequently, a blood sample was collected under deep anesthesia by ketamine (Rotexmedica-ET13L087–11) and xylazine (Alfasan-1801015–18) with a dose of 60 and 20 mg/kg, respectively. The serum was stored at −80 °C after centrifugation at 3000 ×g for 15 min at 4°C to separate the serum for determining serum nitrite levels. Then, the prefrontal cortex (PFC) was extracted from the brain and stored at −80°C to assess nitrite levels and the expression of *iNOS* and *nNOS* genes (Dehkordi et al., 2023).

### 2.3 Induction of seizures and evaluation of the seizure threshold

To induce seizures, an intravenous injection of PTZ (Sigma-P6500) was administered. PTZ (0.5 % solution) was infused into the tail vein of freely moving mice using a 30-gauge dental needle connected by polyethylene tubing to a Hamilton microsyringe. PTZ was injected at a rate of one milliliter per minute using a seizure pump. The onset of a general clonus was used as the endpoint. The general clonus was characterized by clonus of all four limbs with transient loss of righting reflex (Haj-Mirzaian et al., 2019). The seizure threshold was calculated by using the time between injection of PTZ and onset of the seizure, and calculated by the following formula:

$$\mathrm{Threshold concentration}(\frac{\mathrm{mg}}{\mathrm{kg}})\;=\frac{\mathrm{Rate}\left(\frac{\mathrm{ml}}{\min}\right)\times \mathrm{Time}(s)\times \mathrm{Concenteration}(\frac{\mathrm{mg}}{\mathrm{ml}})\times 1000}{60\times \mathrm{Body weight of animal}}$$

### 2.4 Determination of nitrite in serum and PFC

The nitrite concentration in the prefrontal cortex and serum samples was analyzed using the Griess reaction. Initially, 100 µl of each serum or homogenized PFC tissue sample was mixed with 100 µl of Griess reagent. After incubating for 10 min at room temperature, the absorbance at 540 nm was measured using an automatic plate reader (LQ-300 + II-Epson, USA). Finally, sodium nitrite standard curves (Sigma, USA) were utilized to calculate the nitrite concentration of each sample (Mumtaz et al., 2022).

### 2.5 Determination of *iNOS* and *nNOS* gene expressions in the PFC

Real-time polymerase chain reaction (RT-PCR) was employed to identify and quantify *iNOS* and *nNOS* gene expression in the PFC. Following the manufacturer's recommendations, total Ribonucleic acid (RNA) was extracted from tissue using the RNX-plus isolation reagent (Sinaclon, Iran). The cDNA Synthesis Kit (Yekta Tajhiz, Iran) was used to create cDNA, following the manufacturer's instructions. Subsequently, RT-PCR was conducted to assess changes in mRNA expression of the target genes using Rotor-Gene 3000 (Qiagen, Hilden, Germany). The thermal cycling procedure involved an initial activation step of 30 s at 96°C, followed by 45 cycles of denaturation for 5 s at 96°C, and a combined annealing and extension step of 20 s at 60 °C. A melting curve analysis was performed to confirm that all primers generated a single PCR product. The Beta-2-microglobulin (B2M) gene was used as a normalizer (Table 1), and alterations in the expression of each desired mRNA were calculated using the 2^-ΔΔCt^ relative expression formula described previously (Arabi et al., 2021).

**Table 1:** Primer sequences.

Primer	Forward **(5′–3′)**	Reverse **(5′–3′)**
*B2M*	TGGTCTTCTGGTCTTGTC	CAGTTCAGTATGTTCGGCTTCC
*iNOS*	CCAACAGGAGAAGGGGACGAA	GGACATCAAAGGTCTCACAGGC
*nNOS*	GGCTGTGCTTTGATGGAGATGA	AGAATAGGAGGAGACGCTGT

### 2.6 Data analysis

Data analysis was performed using Prism software (version 8). The normality of data was evaluated using the Shapiro-Wilk test. The obtained data were analyzed by one-way analysis of variance (ANOVA) followed by Tukey's post hoc test. *p* < 0.05 was considered statistically significant, and the results were reported as the mean ± standard deviation (SD). The sample size was calculated using G*Power software (ver. 3.1.7). With α set at 0.05 and power (1 − β) at 0.8, the required sample size was six animals per group for behavioral tests and four samples per group for biochemical and molecular assessments.

## 3 Results

### 3.1 GER increased the seizure threshold

**Fig. 2:**
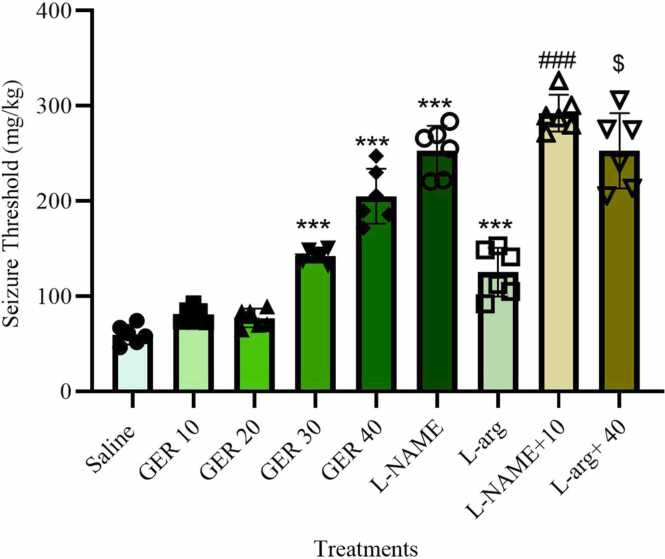
Effect of GER and co-treatments with L-arg and L-NAME on the seizure threshold of PTZ-induced seizures in NMRI mice. GER was administered ip at doses of 10, 20, 30, and 40 mg/kg. L-arg (150 mg/kg) and L-NAME (10 mg/kg) were also administered ip. PTZ (0.5 % solution) was infused intravenously to induce seizures. Data represent mean±SD (n = 6). Statistical analysis was performed using one-way ANOVA followed by Tukey's post hoc test. ***p < 0.001 vs. Saline group, ###p < 0.001 vs. 10 mg/kg GER group, $p < 0.05 vs. 40 mg/kg GER group, Saline: normal saline.

The seizure threshold exhibited significant differences among experimental groups (F_8,45_ = 93.8, p < 0.001, η² = 0.9435, 95 % CI [0.848, 1.0]). Administration of GER at doses of 30 and 40 mg/kg, as well as 10 mg/kg of L-NAME, significantly increased the seizure threshold compared to the saline-treated group (*p* < 0.001). Interestingly, administration of L-arg alone also significantly increased the seizure threshold compared to the saline group (*p* < 0.001). Moreover, co-administration of L-arg with 40 mg/kg GER further enhanced the seizure threshold compared to 40 mg/kg GER alone (p < 0.05). Furthermore, the combination of L-NAME with 10 mg/kg GER significantly elevated the seizure threshold compared to the group receiving 10 mg/kg GER alone (p < 0.001) (Fig. 2).

### 3.2 GER decreased nitrite levels in serum

Serum nitrite levels showed significant variation among the experimental groups (F_9,30_ = 94.94, *p* < 0.001, η² = 0.9661, 95 % CI [0.8746, 1.0]). PTZ administration markedly elevated serum nitrite levels compared to the control group (*p* < 0.001). Treatment with GER at doses of 20, 30, and 40 mg/kg significantly reduced nitrite concentrations compared to the PTZ-saline group (*p* < 0.001). The 10 mg/kg GER dose, however, did not produce a significant change in serum nitrite levels compared to the PTZ-saline group.

Administration of the NOS inhibitor L-NAME (10 mg/kg) significantly decreased serum nitrite levels compared to the PTZ-saline group (*p* < 0.001). Co-treatment with L-NAME and 10 mg/kg GER further reduced nitrite levels compared to 10 mg/kg GER alone (*p* < 0.001).

**Fig. 3:**
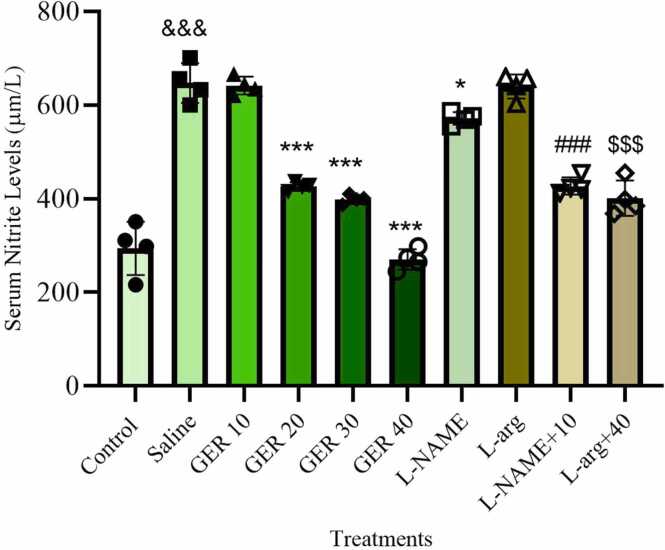
Effect of GER and co-treatments with L-arg and L-NAME on serum nitrite levels in NMRI mice. GER was administered ip at doses of 10, 20, 30, and 40 mg/kg. L-arg (150 mg/kg) and L-NAME (10 mg/kg) were also administered ip. PTZ (0.5 % solution) was infused intravenously to induce seizures except in the Control group. Serum nitrite levels were determined using the Griess reaction and are expressed in µM. Data represent mean±SD (n = 4 per group). Statistical analysis was performed using one-way ANOVA followed by Tukey's post hoc test. & & &p < 0.001 vs. control group, ***p < 0.001 and *p < 0.01 vs. saline group, ###p < 0.001 vs. 10 mg/kg GER group, $$$p < 0.001 vs. 40 mg/kg GER group, Saline: normal saline.

L-arg alone slightly increased serum nitrite levels compared to the PTZ-saline group, although this increase was not statistically significant. However, when combined with 40 mg/kg GER, L-arg significantly increased serum nitrite levels compared to the group that received 40 mg/kg GER alone (*p* < 0.001) (Fig. 3).

### 3.3 GER decreased nitrite levels in the PFC

**Fig. 4:**
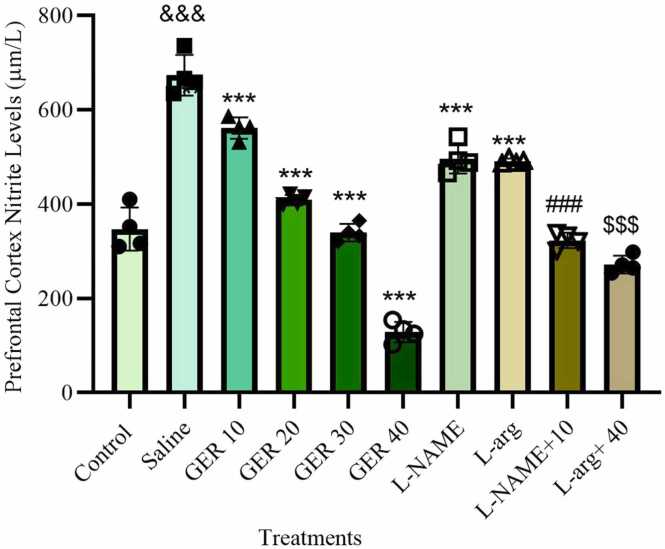
Effect of GER and co-treatments with L-arg and L-NAME on nitrite levels in the prefrontal cortex (PFC) of NMRI mice. GER was administered ip at doses of 10, 20, 30, and 40 mg/kg. L-arg (150 mg/kg) and L-NAME (10 mg/kg) were also administered ip. PTZ (0.5 % solution) was infused intravenously to induce seizures except in the Control group. Nitrite levels in PFC tissue were determined using the Griess reaction and are expressed in µM. Data are presented as mean±SD (n = 4 per group). Statistical analysis was performed using one-way ANOVA followed by Tukey's post hoc test. & & &p < 0.001 vs. control group, ***p < 0.001 vs. Saline group, ###p < 0.001 vs. 10 mg/kg GER group, $$$p < 0.001 vs. 40 mg/kg GER group, Saline: normal saline.

The data provided show significant differences in PFC nitrite levels across groups (F9,30 = 137.7, p < 0.0001, η² = 0.9764, 95 % CI [0.8996, 1.0]). In the saline group, PTZ significantly increased PFC nitrite levels compared to the control group (*p* < 0.001). GER at doses of 10, 20, 30, and 40 mg/kg significantly decreased nitrite levels compared to the control group (*p* < 0.001) (Fig. 4).

L-NAME significantly reduced PFC nitrite levels compared to the control group (*p* < 0.001). Although L-arg is a known precursor of NO, L-arg significantly decreased PFC nitrite levels compared to the control group (p < 0.001).

The combination L-NAME + GER 10 mg/kg significantly further reduced nitrite levels compared to GER 10 mg/kg alone (*p* < 0.001). When L-arg was combined with GER 40 mg/kg, PFC nitrite levels significantly increased compared to GER 40 mg/kg alone (*p* < 0.001).

### 3.4 The expression of *iNOS* and *nNOS* genes in the PFC

**Fig. 5:**
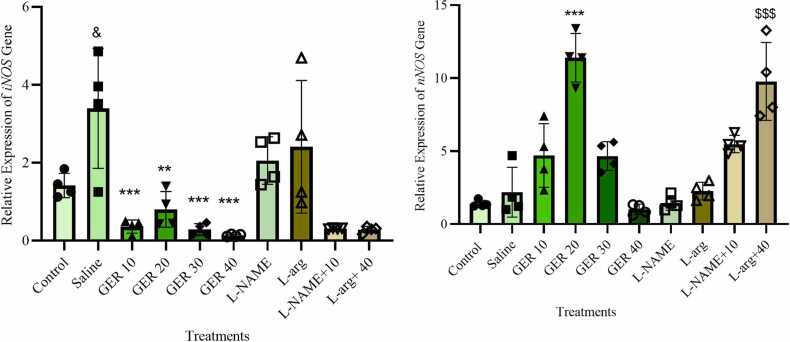
Effect of GER and co-treatments with L-arg and L-NAME on the expression of iNOS and nNOS genes in the prefrontal cortex (PFC) of NMRI mice. GER was administered ip at doses of 10, 20, 30, and 40 mg/kg. L-arg (150 mg/kg) and L-NAME (10 mg/kg) were also administered ip. PTZ (0.5 % solution) was infused intravenously to induce seizures except in the Control group. PTZ-induced changes in iNOS and nNOS gene expression were analyzed using RT-PCR, with B2M as the reference gene. Data are presented as mean±SD (n = 4 per group). Statistical analysis was performed using one-way ANOVA followed by Tukey's post hoc test. &p < 0.05 vs. control group, ***p < 0.001 and **p < 0.01 vs. Saline group, $$$p < 0.01 vs. 40 mg/kg GER group, Saline: normal saline.

The expression of *iNOS* and *nNOS* genes in the PFC exhibited that there are significant differences among experimental groups (F_9,28_=7.983, *p* < 0.001, η² = 0.7188, 95 % CI [0.4912, 0.9464]) and F_9,30_= 26.64, *p* < 0.001, η² = 0.8888, 95 % CI [0.7296, 1.0], respectively). PTZ treatment increased the expression of *the iNOS gene in the PFC compared to the control group (p* < 0.05). The administration of GER at all doses (10, 20, 30, and 40 mg/kg) resulted in a substantial reduction in the expression of the *iNOS* gene compared to the PTZ group (*p* < 0.001). On the other hand, administering GER at a dose of 20 mg/kg resulted in a rise in the expression of the *nNOS* gene compared to the PTZ group (*p* < 0.05). L-arg, L-NAME, and L-NAME plus 10 mg/kg GER did not affect the expression of the *nNOS* and *iNOS* genes in the PFC compared to the PTZ group. Nonetheless, L-arg plus 40 mg/kg GER increased *nNOS* gene expression considerably compared to the group receiving only 40 mg/kg GER (*p* < 0.001) (Fig. 5).

## 4 Discussion

This study investigated the anticonvulsant properties of GER in a PTZ-induced seizure model in mice, with a focus on its modulation of the nitrergic system. The nitrergic system plays a pivotal role in seizure pathophysiology, serving as a critical target for novel antiepileptic interventions. Our findings reveal that GER significantly increases seizure threshold, suppresses NO production, and differentially regulates the expression of *iNOS* and *nNOS* genes in the prefrontal cortex.

NO has been extensively studied for its role in seizure control, exhibiting both neuroprotective and neurotoxic properties depending on the context. Early research has shown that NO produced by *nNOS* acts as a neuromodulator of neuronal excitability and seizure susceptibility, with increased NO levels implicated in epileptogenesis and neuroinflammation (Calabrese et al., 2007, Szabó, 1996). Furthermore, *iNOS*-derived NO is involved in neuroinflammatory processes that exacerbate seizure activity, making it a potential therapeutic target (Gholami et al., 2020, Tawfik et al., 2018). These findings underscore the rationale for exploring NO pathway modulation as a strategy for seizure management, which guided the design of the current study.

The present findings provide compelling evidence that GER significantly increases the seizure threshold in a dose-dependent manner, with the highest efficacy observed at a dose of 40 mg/kg. These data align with the emerging literature; for example, Younis et al. (2024) demonstrated that GER reduces PTZ-kindled seizures in mice by enhancing GABAergic transmission and mitigating neuroinflammation. In their study, GER increased GABA levels and lowered pro-inflammatory cytokines (TNF-α, IL-1β) in neural cells, suggesting a multi-modal anticonvulsant mechanism (Younis et al., 2024). Similarly, Rajendran et al. (2024) found that GER improves cognition in an aging model by reducing oxidative stress and inflammation (Rajendran et al., 2024). Thus, our seizure-threshold results support the concept that GER exerts anticonvulsant effects through both anti-inflammatory and antioxidant actions, as well as the enhancement of inhibitory neurotransmission.

These anticonvulsant effects were further potentiated by co-administration with nitric oxide modulators, suggesting a mechanistic interplay between GER and the NO signaling pathway. Notably, the combination of GER with the NOS inhibitor L-NAME led to a marked elevation in seizure threshold, suggesting that NOS inhibition amplifies the anticonvulsant effect of GER. While L-arg, a precursor of NO, also independently increased seizure threshold, suggesting a potential modulatory impact of L-arg on seizure susceptibility. Additionally, it enhanced GER's effects at the highest dose tested, although with complex interactions, suggesting a synergistic interaction. NO plays a complex role in seizure pathophysiology; while low physiological levels may exert neuroprotective effects, excessive NO production has been implicated in seizure exacerbation and neuronal damage (Rajasekaran, 2005, Theard et al., 1995). The observation that L-NAME, a non-selective NOS inhibitor, significantly enhanced GER's anticonvulsant effect suggests that inhibition of excessive NO production may be a key mechanism through which GER exerts its protective actions. The current study expands upon these findings by highlighting the involvement of the nitric oxide system in GER's anticonvulsant mechanism.

As the results showed, PTZ may increase the production or release of NO, possibly as part of a seizure-related response. In line with this, GER was able to reduce nitrite levels in serum and PFC significantly. This consistent reduction across all doses indicates GER likely inhibits NO production or affects its downstream metabolism, with a dose-dependent effect implied by the range. This effect was potentiated by L-NAME, indicating effective suppression of nitric oxide synthesis. Co-treatment with L-NAME and 10 mg/kg GER further reduced nitrite levels compared to 10 mg/kg GER alone, highlighting a synergistic interaction in reducing NO production. This suggests that the potential inhibitory effect observed with 10 mg/kg GER is enhanced in the presence of L-NAME, possibly indicating that both act on the same pathway.

While L-arg combined with 40 mg/kg GER increased nitrite levels, suggesting a dose-dependent modulation of NO pathways. This indicates L-arg counteracts GER's inhibitory effect, likely by providing more substrate for NO production, overcoming GER's reduction at this effective dose. These results align with studies showing that antiepileptic agents, such as levetiracetam, reduce brain nitrite levels (de Souza et al., 2019). Additionally, GER's ability to decrease nitrite levels is consistent with its anticonvulsant effects observed in other studies involving essential oils rich in GER, such as *Cymbopogon winterianus* and *Ocimum gratissimum* (Freire et al., 2006, Quintans-Júnior et al., 2008).

The differential effects of L-arg in combination with GER on seizure threshold and NO production can be explained by its dose-dependent role in modulating the nitrergic pathway. In PTZ-induced models, L-arg increases nitric oxide levels, which may counteract GER's inhibitory effect on nitric oxide synthesis, leading to an attenuation of its anticonvulsant properties. This dose-dependent duality is consistent with previous studies demonstrating both proconvulsant and anticonvulsant effects of L-arg based on the induction model and co-administration with other agents (Nidhi et al., 1999). Thus, while L-arg slightly increased the seizure threshold when co-administered with GER, it simultaneously reversed its neuroprotective effects by enhancing NO levels. However, further studies are needed to definitively resolve this controversy.

Regarding *NOS* gene expression, GER downregulated *iNOS* expression across all doses (10–40 mg/kg), counteracting the PTZ-induced increase in *iNOS*. This suppression is likely linked to GER's anti-inflammatory and antioxidant properties, as iNOS-derived NO is implicated in neuroinflammatory processes (Tawfik et al., 2018). GER's ability to suppress inflammation through pathways such as *NF-κB* and MAPK signaling (Wu et al., 2020). further supports its role in mitigating seizure-related inflammation. In contrast, *nNOS* expression exhibited a dose-dependent response, increasing at 20 mg/kg GER but decreasing at 40 mg/kg. This apparent contradiction may be attributed to post-translational regulatory mechanisms that inhibit *nNOS* activity despite increased transcription. Additionally, feedback inhibition triggered by elevated NO levels or enhanced NO scavenging could account for the observed reduction in nitrite. Further studies are required to elucidate these regulatory mechanisms and their implications for GER's anticonvulsant effects. Although this observation can be justified through several biological mechanisms, it is recommended that other researchers replicate this finding to confirm its reliability and consistency. Co-administration of L-arg with 40 mg/kg GER increased *nNOS* expression and seizure threshold, suggesting that *nNOS*-derived NO may play a protective role under specific conditions (Su et al., 2014). This dual role of nNOS is consistent with previous research indicating its involvement in both convulsive and anticonvulsive effects, depending on the context (Watanabe et al., 2013).

Beyond the nitrergic system, GER's anticonvulsant effects may involve additional mechanisms. For instance, GER has been shown to enhance GABAergic transmission, as evidenced by its role in increasing inhibitory inputs in other brain regions (Xu et al., 2022). This suggests a potential complementary pathway through which GER exerts its protective effects. Furthermore, GER's antioxidant and anti-inflammatory properties, as demonstrated in other studies (Hacke et al., 2021; Kandeil et al., 2019), likely contribute to its ability to reduce central nervous system excitability and neuroinflammation, further supporting its therapeutic potential.

The safety profile of GER is also noteworthy. The doses used in this study (10–40 mg/kg) are well below the reported median lethal dose (LD_50_) of 4.8 g/kg (gavage) in rats and 4 g/kg (*intramuscular*) in mice (Lapczynski et al., 2008), indicating its potential as a safe therapeutic candidate.

However, this study has several limitations. First, it relied solely on the PTZ-induced seizure model, which primarily reflects acute myoclonic and generalized tonic-clonic seizures. While PTZ is well-established for assessing seizure threshold and acute anticonvulsant effects, it does not fully replicate the pathophysiological features of chronic epilepsy or focal seizures, such as those induced by kainic acid, pilocarpine, or kindling models. These models are known to mimic long-term neuroplastic changes, spontaneous recurrent seizures, and hippocampal sclerosis—key symbols of chronic epileptic conditions. To confirm the generalizability of GER's effects, future studies should employ chronic epilepsy models such as kindling, kainic acid, or pilocarpine, which better capture the complexity and clinical challenges of long-term epileptic conditions.

Second, the molecular and biochemical analyses were limited to the PFC. While the PFC is critically involved in the modulation of neuronal excitability and nitric oxide pathways, other regions such as the hippocampus and amygdala, which are involved in the pathophysiology of seizure, were not examined. Future studies are warranted to explore these additional brain regions, providing a more comprehensive understanding of GER's anticonvulsant mechanisms. Third, this study focused exclusively on the nitrergic system, leaving the contributions of different pathways (such as the GABAergic and glutamatergic systems) unknown, despite evidence of broader neuroprotective effects of GER. Fourth, acute administration of GER limits insight into its long-term efficacy or potential toxicity in repeated-dose scenarios. Finally, the experiments were performed only on male mice, which may not account for sex differences or species variation in seizure responses.

These results suggest that GER exerts its protective effects through intricate modulation of the NO pathway, potentially positioning it as a candidate for seizure management.

## 5 Conclusions

In conclusion, this study demonstrates that GER exhibits anticonvulsant properties through modulation of the nitrergic system, particularly by downregulating *iNOS* expression and reducing NO production. These findings, combined with GER's broader neuroprotective effects, highlight its potential as a novel therapeutic agent for seizure management. Further research is warranted to explore its long-term efficacy, safety, and mechanisms in diverse seizure models and populations.

## Funding

This work was supported by grant No. 4008 from the Research Council of Shahrekord University of Medical Sciences. A. Sureda was supported by the Spanish Government, 10.13039/501100004587Institute of Health Carlos III (CIBEROBN CB12/03/30038).

## CRediT authorship contribution statement

**Dehkordi Hossein:** Writing – review & editing, Writing – original draft, Methodology, Investigation. **Hossein Amini-Khoei:** Writing – review & editing, Writing – original draft, Project administration, Formal analysis, Data curation, Conceptualization. **Antoni Sureda:** Writing – review & editing, Writing – original draft, Validation, Supervision, Methodology, Funding acquisition. **Mehrdad Shahrani:** Writing – review & editing, Writing – original draft, Visualization. **Zahra Lorigooini:** Writing – review & editing, Writing – original draft, Validation, Supervision, Project administration, Methodology, Funding acquisition, Conceptualization. **Babak Shahhosseini:** Writing – review & editing, Writing – original draft, Methodology, Investigation, Conceptualization.

## Declaration of Competing Interest

The authors declare that they have no known competing financial interests or personal relationships that could have appeared to influence the work reported in this paper.

## Data Availability

The data that support the findings of this study are available from the corresponding author upon reasonable request [z.lorigooini@gmail.com].
